# Imaging of inner ear malformations: a primer for radiologists

**DOI:** 10.1007/s11547-021-01387-z

**Published:** 2021-07-01

**Authors:** Paola Feraco, Silvia Piccinini, Cesare Gagliardo

**Affiliations:** 1 Neuroradiology Unit, Ospedale S. Chiara, Azienda Provinciale Per I Servizi Sanitari, largo medaglie d’oro 9, 38122 Trento, Italy; 2 Department of Experimental, Diagnostic and Specialty Medicine, University of Bologna, Bologna, Italy; 3 Neuroradiology Unit, University Hospital of Modena, Modena, Italy; 4 Section of Radiological Sciences, Department of Biomedicine, Neurosciences and Advanced Diagnostics, University of Palermo, Palermo, Italy

**Keywords:** Inner ear malformations, Sensorineural hearing loss, Multidetector Computed Tomography, Magnetic resonance imaging

## Abstract

In the multidisciplinary management of patients with inner ear malformations (IEMs), the correct diagnosis makes the differences in terms of clinical and surgical treatment. The complex anatomical landscape of the inner ear, comprising several small structures, makes imaging of this region particularly challenging for general radiologists. Imaging techniques are important for identifying the presence and defining the type of IEM and the cochlear nerve condition. High-resolution magnetic resonance imaging (MRI) sequences and high-resolution computed tomography (HRCT) are the mainstay imaging techniques in this area. Dedicated MRI and HRCT protocols play an important role in the diagnosis and treatment of patients with inner ear disease. The most suitable technique should be selected depending on the clinical setting. However, in cases of congenital malformation of the inner ear, these techniques should be considered complementary. Since prompt intervention has a positive impact on the treatment outcomes, early diagnosis of IEMs is very important in the management of deaf patients. This article reviews the key concepts of IEMs for clinical radiologists by focusing on recent literature updates, discusses the principal imaging findings and clinical implications for every IEM subgroup, thus providing a practical diagnostic approach.

## Introduction

Inner ear malformations (IEMs) are caused by interrupted development of the ear during the first trimester of foetal development and are the principal cause of sensorineural hearing loss (SNHL) [1].

Imaging techniques have a long history in supporting the study of the auditory system. However, the anatomy and related pathology of the auditory system necessitate the use of specific, dedicated, optimised protocols to ensure good visualisation. In particular, computerised tomography (CT) and magnetic resonance imaging (MRI) are the most commonly used imaging modalities for investigating the auditory system at any level. High-resolution CT is the preferred imaging modality for delineating the osseous anatomy and malformations of the inner ear. However, in the last two decades, MRI has been widely used to study endolymph-filled structures and the eighth cranial nerve (i.e. the vestibulocochlear nerve) [1, 2].

Inner ear malformations can be associated with both hereditary and nonhereditary SNHL. Indeed the aetiology of IEM can be idiopathic, related to an inborn genetic error, or in response to exposure to a teratogenic agent [2]. Although IEMs may occur in isolation, around 30% of these cases are related to a specific syndrome [3, 4] (Table 1). Patients with genetic IEMs usually have bilateral and symmetrical abnormalities and are commonly affected by profound SNHL. Since prompt intervention has a positive impact on the treatment outcomes, early diagnosis of IEMs is very important in the management of deaf patients.

**Table 1:** Principal hereditary syndromes commonly associated with SNHL and IEMs (see references 4,6,35) and their specific related imaging findings. PSCC: posterior semicircular canal; LSCC: lateral semicircular canal; IAC: internal auditory canal; IP-III: incomplete partitions type III

Hereditary syndrome associated with SNHL	Related imaging findings
Alagille syndrome	Absence of the PSCC with normal-appearing LSCC
Branchio-oto-renal syndrome	Cochlear hypoplasia (apical turn); abnormal course of the facial nerve canal; funnel-shaped IAC with large porus acousticus; vestibular dysplasia; SCC hypoplasia; enlargement of the vestibular aqueduct; cochlear nerve deficiency
CHARGE syndrome	SCCs aplasia with associated vestibular dysplasia; cochlear nerve deficiency with atresia of the cochlear aperture; abnormalities of cochlear partitioning
Pendred syndrome	Modiolar deficiency; vestibular enlargement; absence of the interscalar septum between the upper and middle cochlear turn; endolymphatic sac enlargement
Waardenburg syndrome	Vestibular aqueduct enlargement; widening of the upper vestibule; IAC hypoplasia; decreased modiolus size; aplasia or hypoplasia of the PSCC
X-linked hearing loss with stapes gusher	Enlarged bulbous IACs; IP-III; widening of the bony canal for the labyrinthine segment of the facial nerve; dilation of the vestibular aqueducts

The aim of this pictorial review was to describe the principal imaging findings for every IEM subgroup, providing a practical diagnostic approach for general radiologists by focusing on recent literature updates. An extensive search of English literature was performed on PubMed (https://pubmed.ncbi.nlm.nih.gov) using the following keywords and their combinations: inner ear malformations, cochlea, MRI, CT, cone-beam.

## Normal anatomy and development of the inner ear

**Fig. 1:**
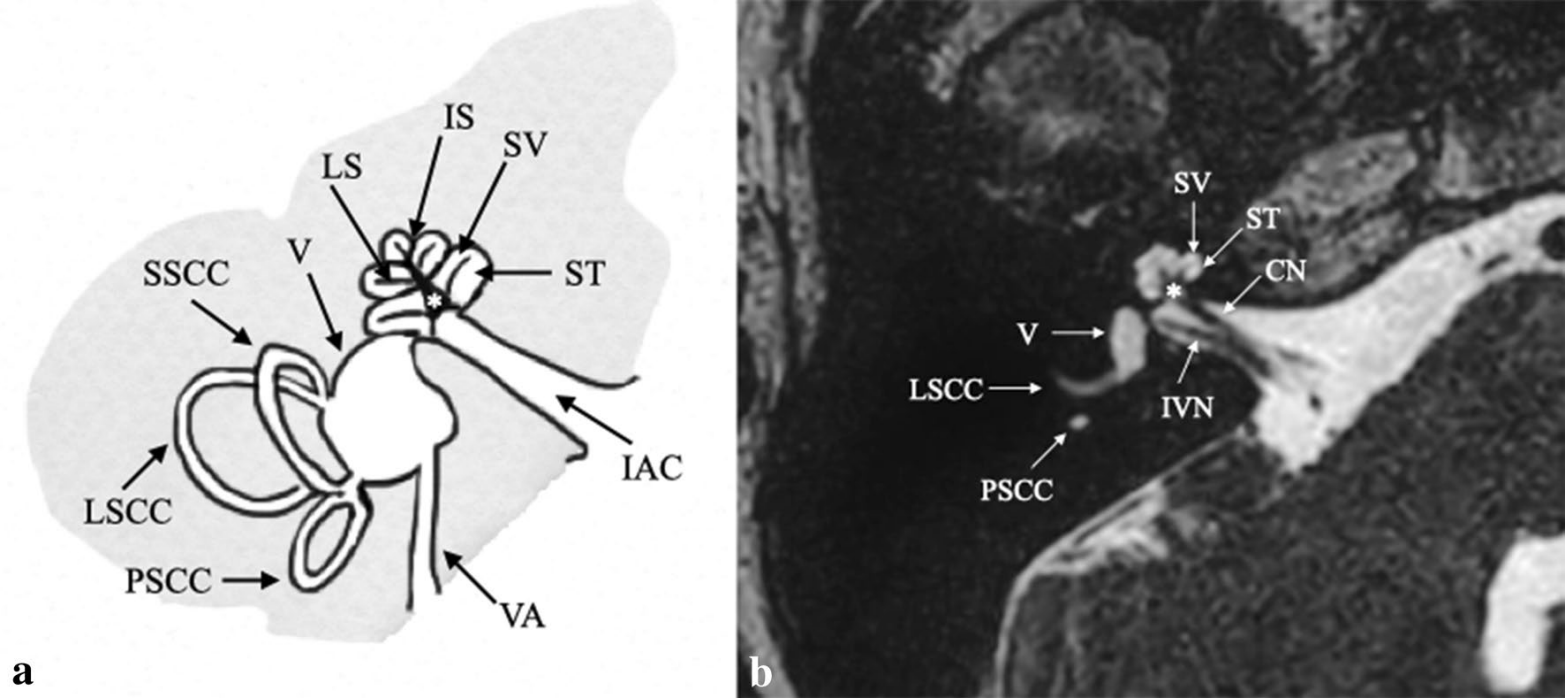
Normal anatomy of Inner Ear structures in drowning (a) and high-resolution 3 T MRI (b) (0.8-mm thick axial MPR from 3D FIESTA sequence). The cochlea consists of a canal that helices 2½ to 2¾ times around a central column of bone (the modiolus, asterisk). The modiolus is the base of the cochlea at the fundus of the internal acoustic canal (IAC). The cochlear spirals are divided by the interscalar septum (IS). A thin osseous spiral lamina (LS) that developments from the modiolus divides the cochlear canal in two compartments: scala vestibuli (SV) and a scala tympani (ST). Legend: CN: cochlear nerve; IVN: inferior vestibular nerve; V: vestibule; LSCC: lateral semicircular canal; PSCC: posterior semicircular canal; SSCC: superior semicircular canal; vestibular aqueduct (VA).

Figure 1 reviews normal inner ear (IE) anatomy. The IE includes the bony labyrinth (otic capsule), which consists of the cochlea, vestibule, semicircular canals (SCCs), vestibular aqueduct, and membranous labyrinth, which includes the utricle, saccule, endolymphatic sac and duct, as well as the cochlear duct. A fluid known as perilymph fills the space between the osseous labyrinth and membranous labyrinth, while the membranous labyrinth is filled with endolymph [5].

The cochlea is responsible for hearing, while the vestibule and SCCs are important for balance.

**Fig. 2:**
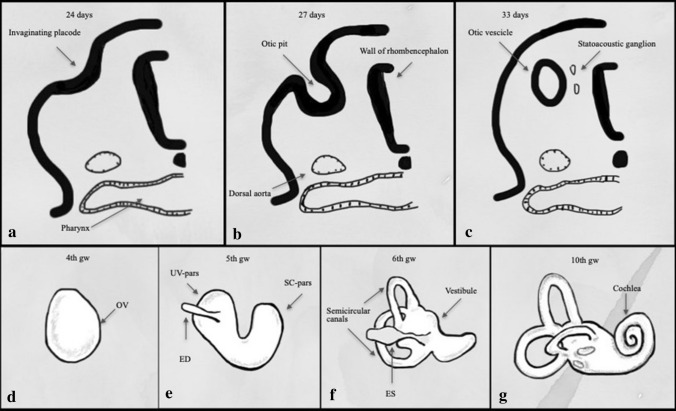
Schematic description of Inner ear development. Transverse section through the rhombencephalon (**a–d**) showing formation of the otic vescicle (OV) by thickening and invagination of the ectoderm (otic placode). In the fifth week (**e**), the otocyst becomes infolded, forming the upper pars utricoluvestibularis (UV) and the lower parssacculocochlearis (SC). In the sixth week (**f**), the three semicircular canals form from the UV-pars. In the seventh to ninth weeks (**g**), the cochlear duct forms as a tubular extension of the SC-pars and becomes coiled

The embryological development of the IE begins with the focal thickening of the ectoderm (otic placode) and proceeds via multiple stages [5–7] from 3rd to 24th gestational week (gw) (Fig. 2):

- 3rd gestational week (gw): otic placodes arise from the surface ectoderm on each side of the rhombencephalon;
- The statoacoustic ganglia cells appear;
- 4th gw: otic placodes invaginate and give rise to the otic pits which, towards the end of the fourth week, form the corresponding otic vescicle (or otocysts) and auditory vesicles; formation of statoacoustic ganglion;
- 5th gw: the otocysts divide in dorsal utricular portion and ventral saccular portion which give rise to the vestibular and cochlear divisions of the labyrinth, respectively; the SSCs start to develop (superior, posterior, and lateral canals consecutively); the statoacoustic ganglion divides
- End of the 6th gw: the membranous cochlea started forming by elongation of anterior aspect of cochlear;
- Division; the SCC is complete; the utricule and saccule are present;
- End of the 7th gw: the maculae are present; sensory ridges appear in the cochlea duct;
- The membranous cochlea completes 2.5 turns. The development of the cochlear lumen starts at the base of the cochlea and continues towards the apex. These changes are partially controlled by fluid secretion in the vestibular labyrinth;
- End of the 8th gw: there are 1.5 cochlear turns;
- 9th gw: saccule connects to the utricule via the ductus runiens; the mesenchyme surrounding the cochlear duct forms cartilage;
- 10th gw: the cochlea obtains nearly adult form and reaches 2.5 turns; the cartilage surrounding the cochlear undergoes vacuolization to form the scala tympany and the scala vestibuli; the original cochlear duct forms the scala media, while a bar of cartilage persists between the scala tympani and the scala vestibuli and forms the modiolus; epithelial cells begin to differentiate into sensory cells of the organ of Corti;
- 11th to 24th gw: thickening of epithelium in the cochlear duct and the labyrinth continues to expand; Ossification of the otic capsule begins (16th gw); The cochlear duct reaches its full length; the membranous labyrinth is full size (20th gw); The otic capsule ossification and the perilymphatic space are complete (23–24th gw).

Otic capsule and inner ear structures are completely formed at birth and remain stable in size over time, except for endolymphatic duct and sac which continues to grow until the age of four years old and the vestibular aqueduct which continues to develop until puberty [5, 8]. An arrest at any one of these stages corresponds to a particular IEM (Table 2).

**Table 2:** Principal characteristics of inner ear malformations (see references 17,19,23). FN: facial nerve; CAVD: cochlear aplasia with a dilated vestibule*;* EVA: enlarged vestibular aqueduct; HA: hearing aid; CI: cochlear implantation; ABI: auditory brainstem implantation

Inner ear malformations	Imaging findings	Facial nerve routes anomaly	Treatment
Complete labyrinthine aplasia	Absent labyrinth (cochlea, SCCs and vestibule)	Yes	ABI
Rudimentary otocyst	Millimetric otic capsule residue	Yes	ABI
Cochlear aplasia	Absent cochlea with different degree of representation of SCCs and vestibule (divided in “CA with normal labyrinth” and “CAVD”)	Yes	ABI
Common cavity	Cochlea and vestibule have a single cavity	Yes	CI or ABI
Cochlear hypoplasia	Small size of the cochlea with various internal structural abnormalities (four types [19])	Yes	HA, CI or ABI
Incomplete partition I	Cochlea with cystic appearance	Possible	CI or ABI
Incomplete partition II	Cystic appearance of the cochlear apex associated with a minimally dilated vestibule and enlarged vestibular aqueduct	No	HA or CI
Incomplete partition III	Modiolus absent, interscalar septa present	Yes	HA or CI
EVA	EVA with normal cochlea	No	HA or CI

## Imaging techniques

The unique anatomy of the auditory system necessitates the use of specific and optimised imaging protocols. In particular, high-resolution CT (HRCT) and multiplanar MRI sequences are widely used for investigating suspected IEMs as they are complementary in the evaluation of cochlear implant candidates [9, 10].

**Fig. 3:**
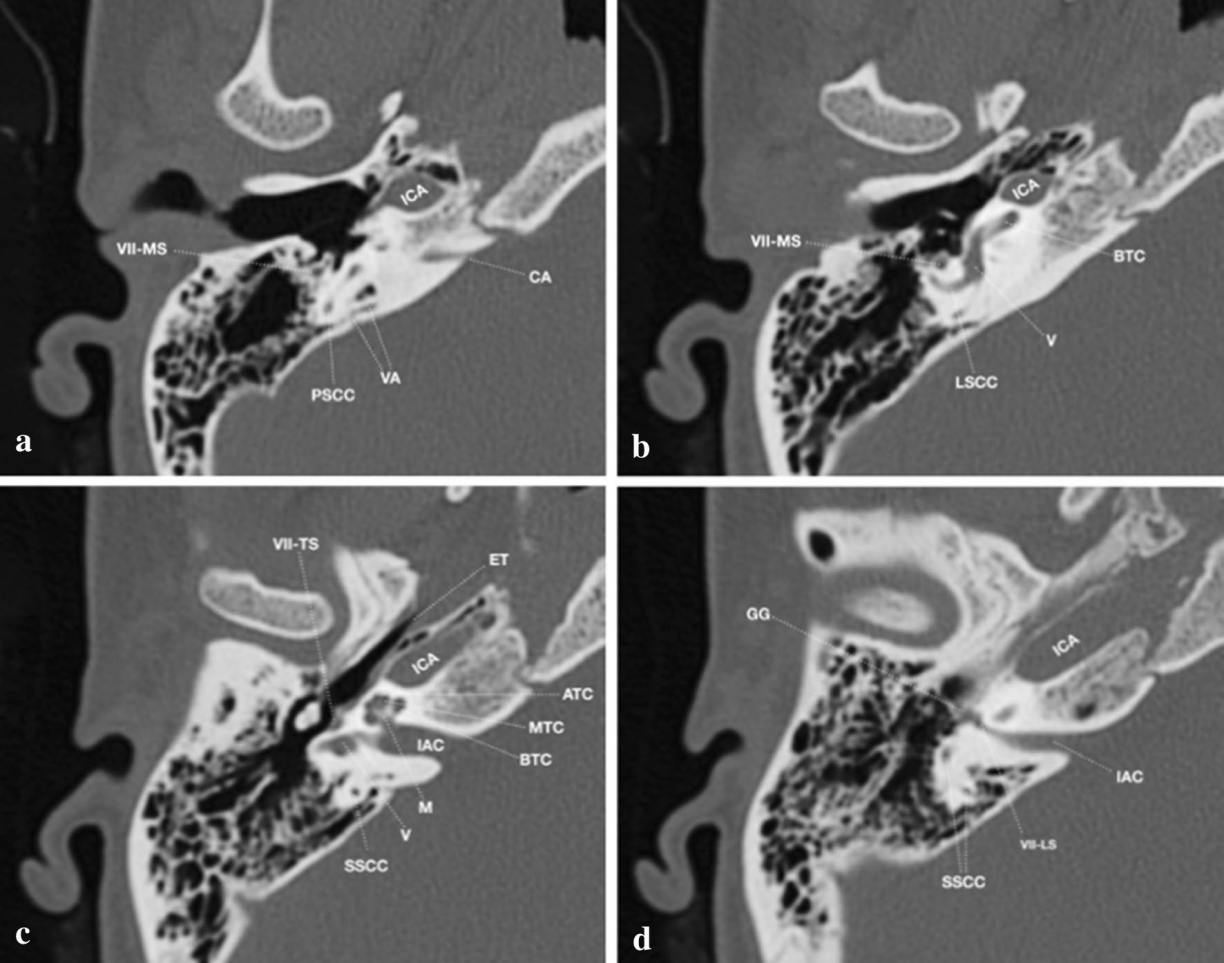
Normal anatomy of Inner Ear structures in high-resolution CT (selection of 0.625-mm thick axial slices); caudocranial direction from **a** to **d**. Legend: ATC: apical turn cochlea; BTC: basal turn cochlea; MTC: middle turn cochlea; CA: cochlear aqueduct; ET: eustachian tube (entrance); IAC: internal auditory canal; ICA: internal carotid artery; M: malleus; LSCC: lateral semicircular canal; PSCC: posterior semicircular canal; SSCC: superior semicircular canal; V: vestibule; VA: vestibular aqueduct; VII: 7th cranial nerve (LS: labyrinthine segment; MS: mastoid segment; TS: tympanic segment); GG: geniculate ganglion

Figure 3 reviews normal temporal bone anatomy on HRCT. HRCT is the preferred imaging modality for delineating the osseous anatomy and IEMs. Temporal bone HRCT, which provides excellent anatomical details, should be performed either in helical scanning mode (0.5–0.625-mm thickness) using the orbitomeatal line as the scanning baseline, or direct axial and coronal CT in conventional sequential scanning mode (0.08–1.25-mm thickness). The use of multidetector CT scanners enables rapid volumetric acquisitions (especially if isotropic data are acquired) that can be used to generate multiplanar reconstructions (0.6 mm thickness). Coronal and sagittal multiplanar reconstructions (MPRs) may aid in precise localization of IEMs. However, although HRCT is the standard imaging method used to assess a patient with hearing loss, recent studies indicate that cone-beam computed tomography (CBCT) may provide an alternative. Indeed, the higher spatial resolution, the fewer metal artefacts production and the lower radiation dose made it a reliable alternative for temporal bone imaging [11]. In particular CBCT, which is clearly useful in the paediatric population, has been proposed for intraoperative evaluation during temporal bone surgery and for precise cochlear implants location [12–14].

On the other hand, MRI is used to study endolymph-filled structures and the eighth cranial nerve (i.e. the vestibulocochlear nerve) [1, 14].

Thin-slice (0.4–0.8-mm thickness) heavily T2-weighted high-resolution 3D fast gradient echo sequences can provide submillimeter assessment of the structure of the fluid-filled inner ear. These sequences are particularly dependent on high gradient amplitude and slew rates and has a cisternographic effect showing very high contrast between liquor and surrounding structures (nerves, vessels, and bone) [6]. The acquired 3D images can be re-formatted in arbitrary orientation because of the sub-millimetric isotropic resolution. 3D MRI reconstructions perpendicular to the internal auditory canal (IAC) and cerebellopontine angle should be provided to better visualise the course of the 7th and 8th nerves. Moreover, the mid-modiolar view needs to be studied to better elucidate the internal cochlear structures, such as the interscalar septum and lamina spiralis.

## Classification

Congenital IEMs are classified into two large groups:

1. Malformations involving only the membranous labyrinth.
2. Malformations of the otic capsule, which involves both the osseous and the membranous labyrinth [15].

Up to 80% of the IEMs causing congenital hearing loss fall in the first group and do not show macroscopic temporal bone abnormalities and related CT and/or MRI changes. On the other hand, the remaining 20% of IEMs can be radiologically detected. Indeed, they involve malformations of the bony labyrinth that can easily be detected and characterised by CT and MRI, together with any associated cranio-encephalic abnormalities.

The first classification of IEMs proposed by Jackler et al. was based on the time of developmental arrest during embryogenesis [16]. In 2002, Sennaroglu and Saatci modified this classification and grouped IEMs based on differences in cochlear anatomy, clinical findings, and treatment options [17]. Subsequently, in 2006, X-linked deafness was recognised as incomplete partition type III and the IEMs classification system was updated [18].

It is very important to correctly classify IEMs with a universally accepted system. In this educational review, we describe the IEMs according to the latest classification system (Table 2), introduced by Sennaroglu et al. [17, 18]. In this classification, similar cochlear anomalies (i.e. those with similar clinical findings and treatment options) are grouped together. This classification is considered the most useful for practical management by surgeons and audiologists. Depending on the type and severity of IEM, patients can be managed by using a hearing aid (HA), cochlear implantation (CI) and, in the most severe cases, auditory brainstem implantation (ABI).

However, when selecting the implantation method, three other elements should be considered along with the IEM classification: cochlear nerve imaging, audiological findings and the risk for cerebrospinal fluid gusher and meningitis.

Finally, since the embryological development of the bony canal of the facial nerve (FN) is closely connected with the development of the IE structures, its related abnormalities are described at the end of each IEM, according to the current literature [19].

## Complete labyrinthine aplasia (Michel deformity)

Complete labyrinthine aplasia (CLA) is a rare IE malformation. It is the most severe of the abnormalities involving the osseous and membranous labyrinth. It seems to result from the developmental arrest of the otic placode [20]. CLA can be classified into three subgroups [21]:

1. *CLA with hypoplastic or aplastic petrous bone*: in this group, CLA is found together with hypoplasia or aplasia of the petrous bone.
2. *CLA without otic capsule*: the otic capsule ranges from hypoplastic to aplastic, while the petrous bone is normal.
3. *CLA with otic capsule*: the petrous bone and the otic capsule are normal.

CT examination of patients with CLA shows evidence of a complete absence of inner ear structures (cochlea, vestibule, and SSCs). The external auditory canal and middle ear cavity are usually normal because they do not arise from the otic capsule [21].

### Key points

- FN: anterior displacement of the labyrinthine segment of the facial canal is seen in CLA, except for CLA with otic capsule, since the otic capsule is developed.
- Audiological findings: patients suffering from CLA present with a complete SNHL.
- Management: since the *vestibulocochlear nerve is aplastic* in CLAs, this group of patients is indicated for ABI rather than CI surgery. Indeed, CLAs are accepted as “Definite Indications for ABI” [22].

### Rudimentary otocyst

**Fig. 4:**
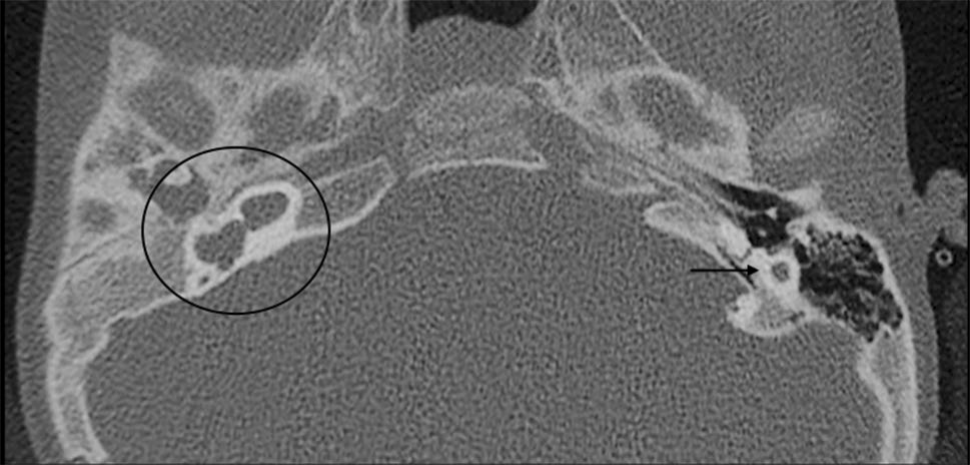
A 2-year-old male with bilateral SN deafness from birth. Axial CT obtained at IAC level shows bilateral atresia of IAC. On the left side the IE structures are absent; a residual otocyst is appreciable (arrow). Formation of the petrous bone is normal but the otic capsule is hypoplastic. On the right side an incomplete partition malformation is also evident (circled)

The term ‘rudimentary otocyst’ is used to describe millimetric representations of the otic capsule without an IAC (Fig. 4) [6]. Some portions of the SCCs may be present. This is a transitional anomaly between Michel deformity and common cavity (CC).

#### Key points

- FN: there is anterior displacement of the labyrinthine segment of the facial canal owing to the small otic capsule.
- Audiological findings: similar to CLA.
- Management: ABI is the only indication for rudimentary otocyst.

### Cochlear aplasia

**Fig. 5:**
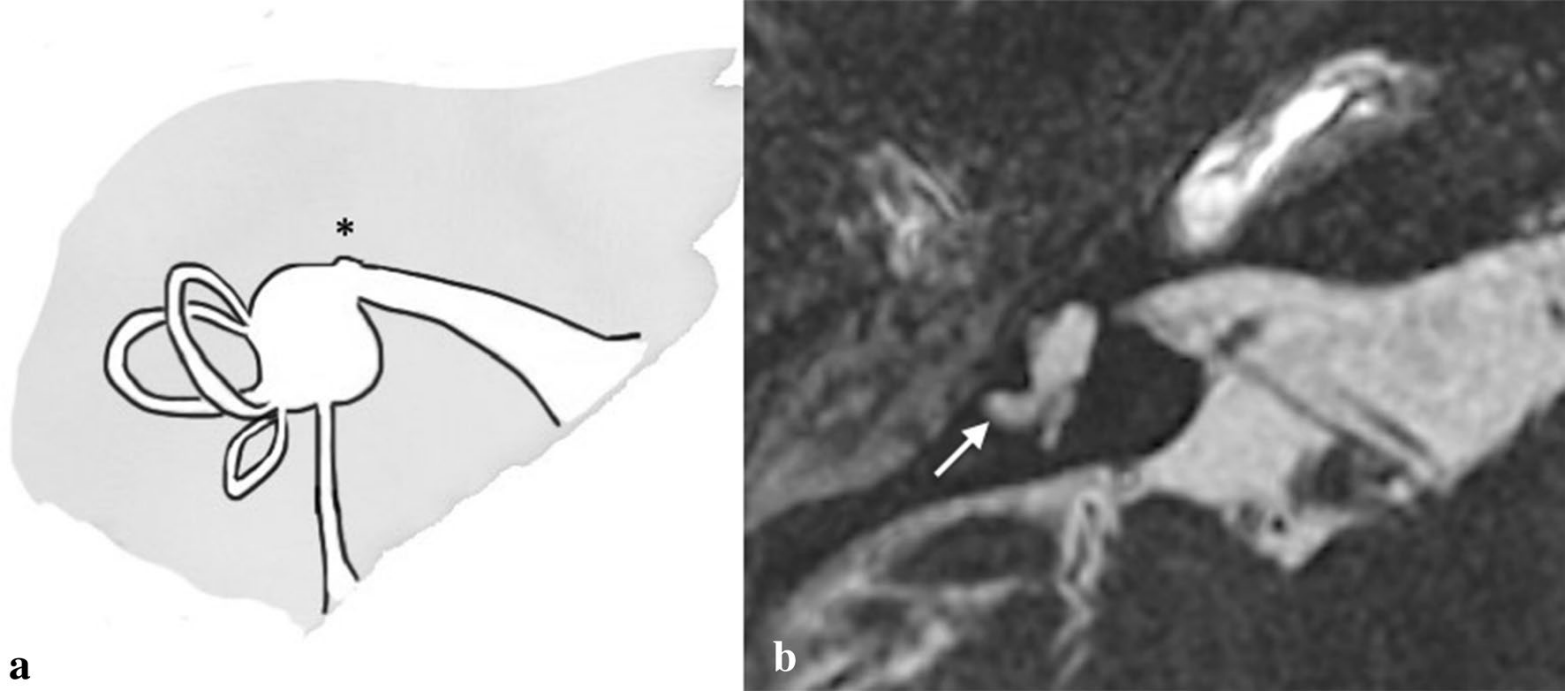
Cochlear aplasia. **a** Drowning shows the total absence of the cochlea (asterisk) and the normal appearance of the IE remaining structures. **b** A 3-year-old female with right SN deafness from birth. Axial 3D FIESTA sequence obtained at IAC level shows cochlear aplasia and dilatation of the vestibule (CAVD, asterisk) and lateral SCC (arrow)

In the cochlear aplasia (CA) group, the cochlea is absent while the utricle, saccule, and semicircular canals are preserved, which can be easily observed by CT scan (Fig. 5). CA can be categorised into two subgroups [23]:

1. *CA with normal labyrinth.*
2. *CA with a dilated vestibule (CADV).*

In the first group, cochlear aplasia is usually symmetric (suggesting a genetic aetiology) while in CADV asymmetric development may be present.

#### Key points

- FN: a moderate anterior dislocation of labyrinthine segment may be detected in CA.
- Audiological findings: complete SNHL.
- Management: the only possible surgical option is ABI.

### Common cavity deformity

**Fig. 6:**
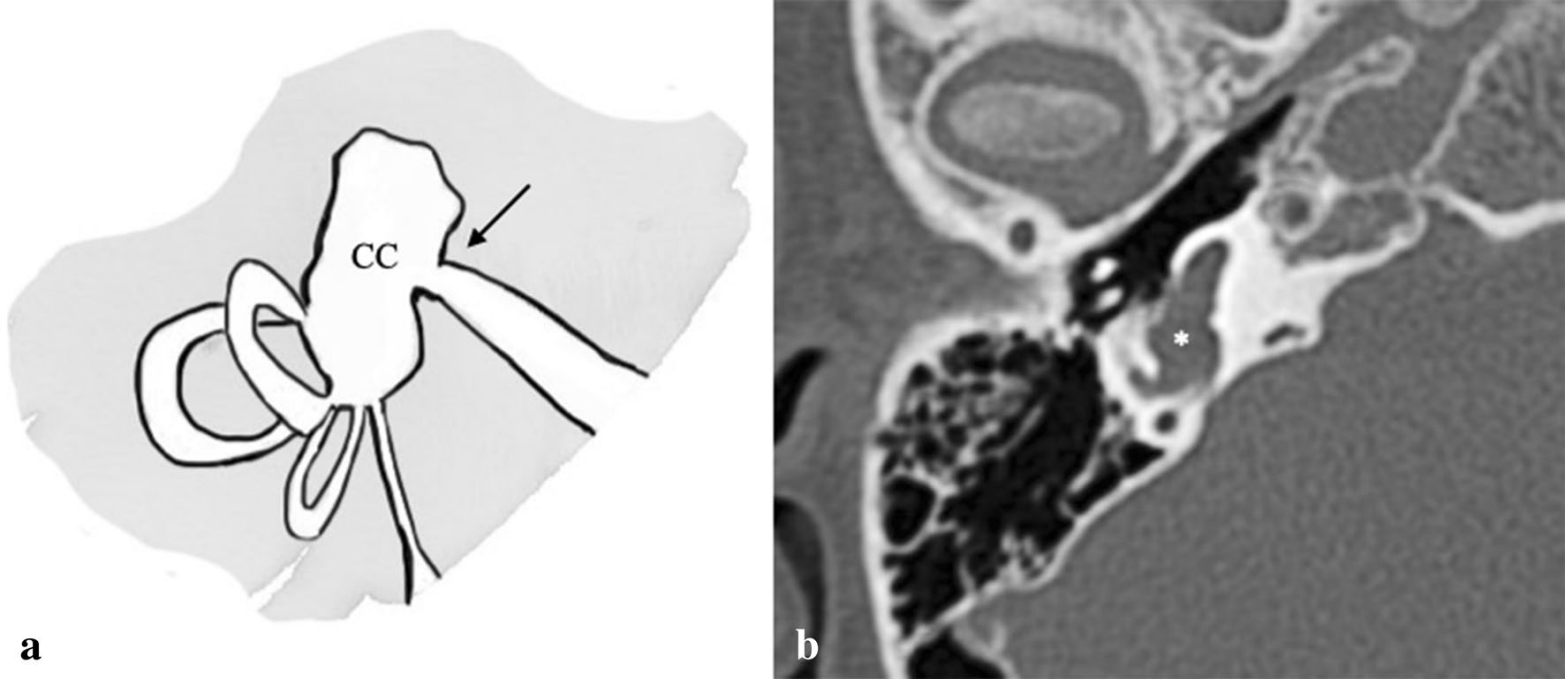
Common cavity. **a** Drowning shows the complete confluence of the vestibule and cochlea (CC) without internal architecture. The fundus of the IAC enters the centre of the CC (arrow); finding that permits to distinguish this malformation from CAVD); **b** A 2-year-old male with bilateral SN deafness from birth. On the right side, temporal bone axial CT scan shows the complete confluence of the vestibule and cochlea (asterisk)

CC deformity accounts for approximately 25% of all cochlear malformations [1]. In the CC deformity, the vestibule and cochlea are confluent with no internal architecture (Fig. 6). The IAC usually enters through the centre of the cavity. There may also be associated SCCs or their undeveloped parts. The principal differential diagnosis includes CAVD.

#### Key points

- FN: anterior displacement of the labyrinthine segment of the facial canal is usually associated. It is important to note that in this malformation *cochlear and vestibular neural structures are present* in varying degrees.
- Audiological findings: profound hearing loss.
- Management: patients who present with CC should undergo to CI.

### Cochlear hypoplasia

**Fig. 7:**
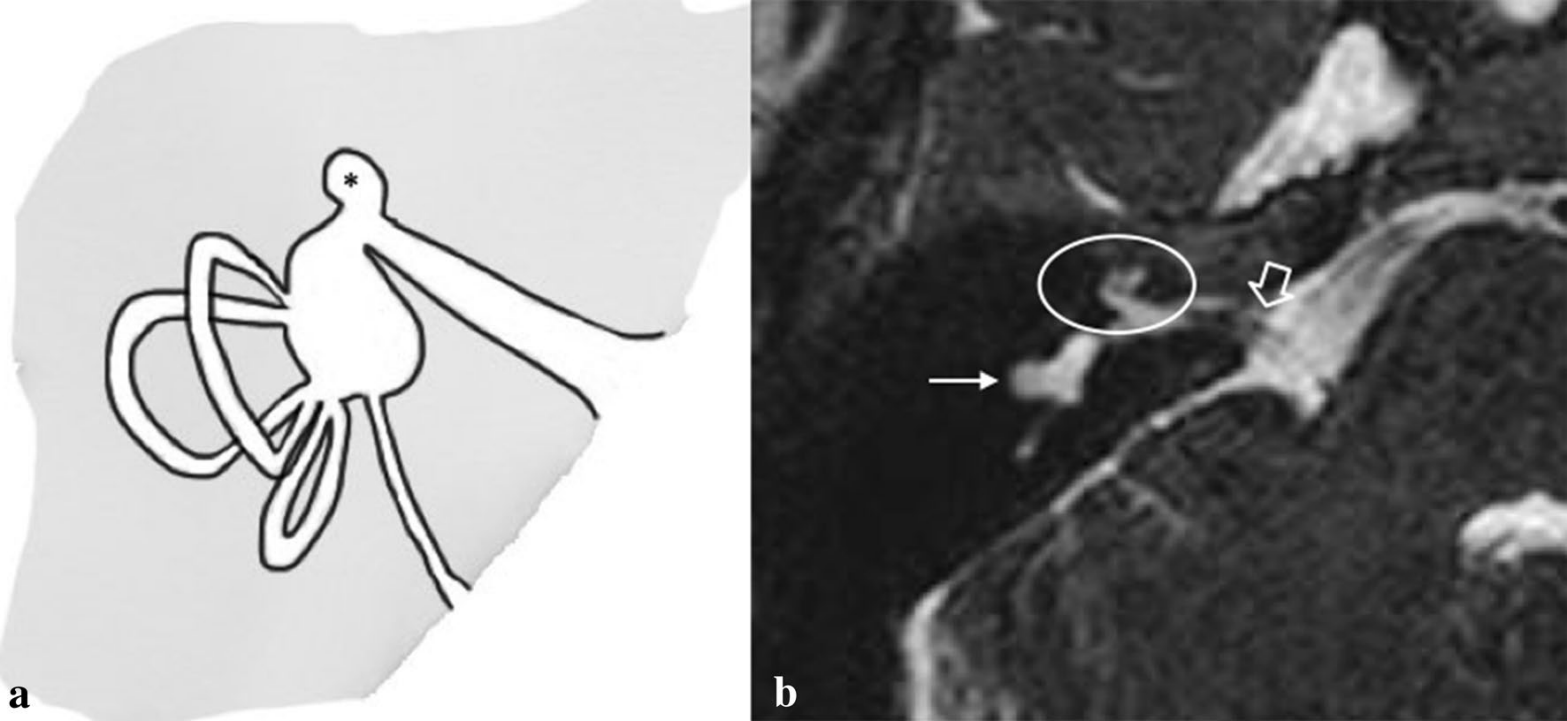
Cochlear hypoplasia. **a** The chart shows type I cochlear aplasia. **b** A 16-year-male with right SN deafness from birth. Axial MRI 3D FIESTA sequence obtained at IAC level shows cochlear hypoplasia (circled), stenosis of the right IAC (open arrow) with cochlear nerve hypoplasia, and LSCC dysplasia (white arrow)

In cochlear hypoplasia (CH), the cochlea is smaller than normal and there is a clear distinction between the cochlea and the vestibule. CT shows a small cochlear bud and an enlarged vestibule. Interestingly, the SSCs are malformed in 50% of CH patients. Moreover, most of the CH displayed narrow IACs (Fig. 7).

CH is divided into four subgroups [24]:

1. *CH-I (bud-like cochlea)*: the internal appearance is deformed; modiolus and interscalar septa cannot be detected.
2. *CH-II (cystic hypoplastic cochlea)*: the cochlea has smaller dimensions with a defective modiolus and interscalar septa, but with a normal external outline.
3. *CH-III (cochlea with less than two turns)*: the modiolus, interscalar septa, and external outline have fewer turns and are smaller than those of a normal cochlea. The vestibule and the SCCs are hypoplastic.
4. *CH-IV (cochlea with hypoplastic middle and apical turns)*.

Since the characterisation of different CH’s subtypes can be challenging, the use of normative values in identifying and classifying CH, rather than rely on visual inspection alone, has been proposed and seems to be promising [8, 25].

#### Key points

- FN: anterior displacement of the labyrinthine segment of the facial canal is usually associated. It is important to note that in this malformation *cochlear and vestibular neural structures are present* in varying degrees.
- CH patients represent the largest group of IEMs in terms of clinical presentation and management.
- Audiological findings: variable SNHL depending on the level of maturity of the membranous labyrinth.
- Management: the options depend on the degree of SNHL and range from hearing aids to ABI (*cochlear nerve deficiency may be observed in patients with CH*). Moreover, some cases of CH (CH-III and CH-IV) may benefit from stapedotomy. Indeed, these groups have pure conductive or mixed hearing loss where the conductive component is due to stapedial fixation.
- Possible complication: in CH-II, the modiolus may be completely absent, creating a wide connection with the IAC; hence, gusher and misplacement of the CI electrode into the IAC are possible. Recurrent meningitis can occur because of a defective stapes footplate.

### Incomplete partitions

**Fig. 8:**
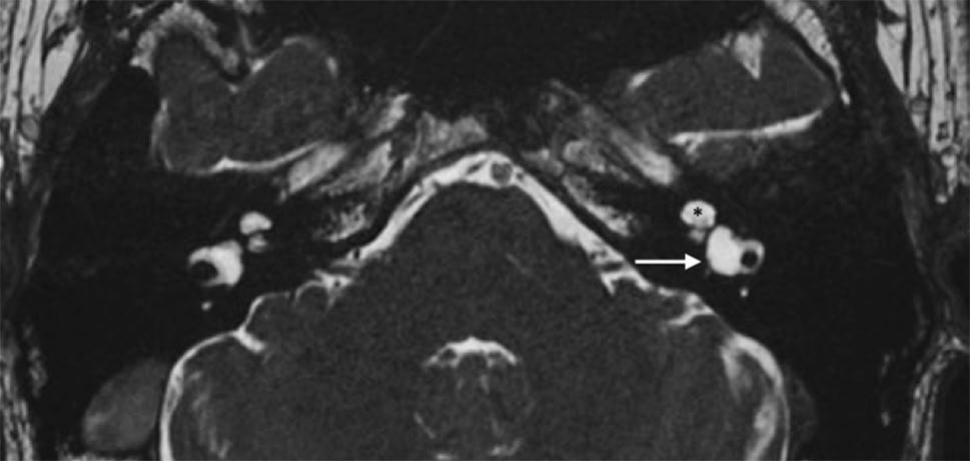
Incomplete partition type I. A 5-year-old female with profound right SNHL. A 45-year-old-man with progressive left HL. Axial MRI T2 high-resolution 3D sequence obtained at IAC level shows typical IP-I malformation abnormalities on the left side (arrow) with a cystic appearance of the cochlea (asterisk) that is clearly separated from the vestibule

**Fig. 9:**
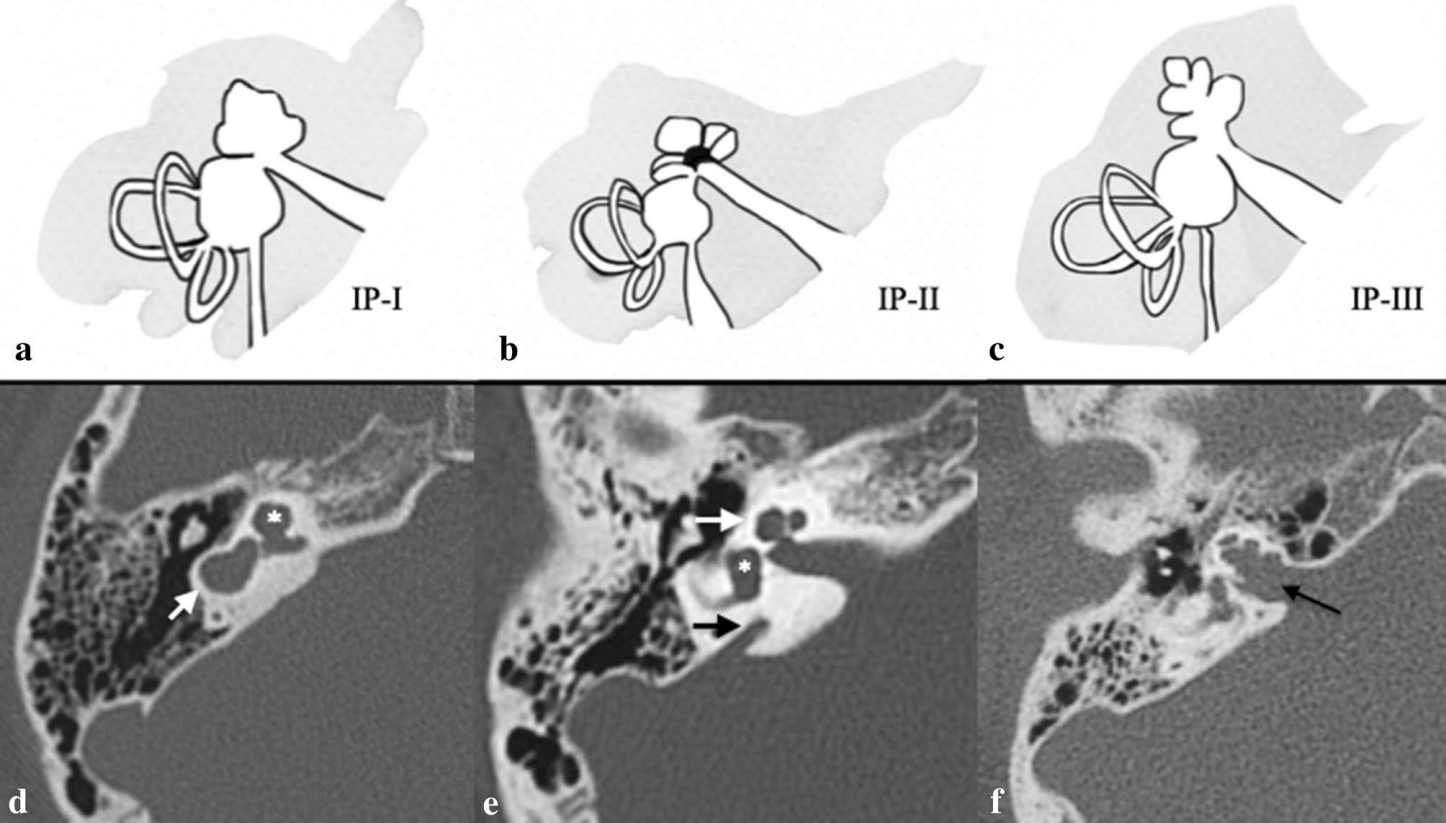
Incomplete partitions. **a–c** Drowning shows schematic representation of IP-I, IP-II and IP-III and their appearance on CT scan (**d–f**). **d** Cystic cochleovestibular malformation (IP-I): clear differentiation between the cochlea (asterisk) and the dilated vestibule (arrow) allow CC to be excluded. **e** Temporal bone axial CT scan of a 6-year-old female with progressive right HL, shows cystic appearance of the upper sections of the cochlea owing to the confluence of middle and apical turns (arrow), dilated vestibule (asterisk), and an enlarged vestibular aqueduct (black arrow): the classic Mondini deformity (IP-II). **f** A 6-year-old male with progressive right HL. Axial CT obtained at IAC level shows assimilation of the cochlea in the bottom part of the IAC in IP-III (arrow)

Incomplete partitions (IP) represent about the 40% of IEMs [23, 24]. In this malformations, there is a clear differentiation between the cochlea and the vestibule. Moreover, the overall proportions are normal but there are various defects in the internal architecture. The IP group is divided into three subgroups depending on the defects in the modiolus and the interscalar septa:

1. *Incomplete partitions type I (IP-I)* (also referred to as “cystic cochleovestibular malformation” [22]). These represent about 20% of IEMs. The cochlea has a cystic appearance owing to the complete absence of the modiolus and interscalar septa. There is a clear differentiation between the cochlea and the vestibule. These findings allow the differentiation of IP-I from CC. Moreover, an enlarged vestibule may be present (Figs. 8, 9).
2. *Incomplete partitions type II (IP-II)* (classic Mondini deformity). This subgroup accounts for more than 50% of all cochlear deformities [15]. The Mondini triad (Fig. 9) comprises:
  - a defect on the apical part of the modiolus;
  - a slightly widened vestibule;
  - an enlarged vestibular aqueduct (EVA) [24, 26].

The apex of the cochlea has a cystic appearance. This is caused by the confluence of the middle and apical turns. The external dimensions of the cochlea are usually normal.

**Fig. 10:**
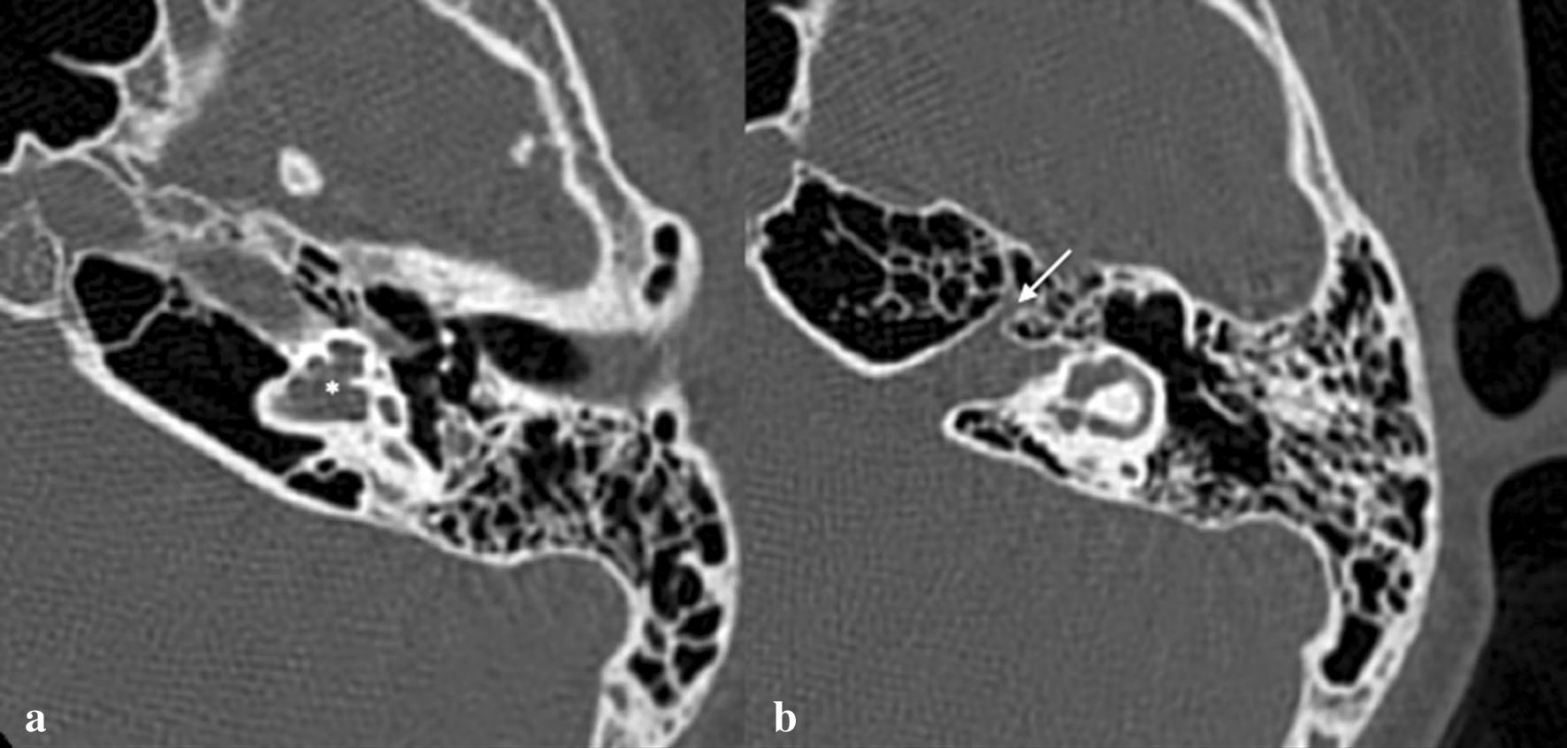
Incomplete partition type-III. Temporal bone axial CT scan of a 12-year-old male with progressive left HL shows an incomplete partition type III (IP-III) (**a**). The interscalar septa is preserved but no modiolus is appreciable (asterisk in **a**). There is an assimilation of the cochlea in the bottom part of the IAC with anteromedial dislocation of the labyrinthine segment of the facial nerve canal (arrow in **b**)

3. *Incomplete partitions type III (IP-III).* This is very rare subgroup accounts from 0.9 to 2% of all IEMs [27, 28]. The cochlea has an interscalar septa but the modiolus is completely absent (Figs. 9 and 10). IP-III cochlear malformation is usually observed in X-linked deafness [17]. It involves not only the cochlea and IAC, but also the whole otic capsule, including the vestibule and SCC [28].

#### Key points

- FN: In IP-I and IP-II, since the cochlear size is normal, the FN has a normal labyrinthine course. On the other hand, superior displacement of the labyrinthine segment of the facial canal is typically seen in IP-III owing to the defective outer periosteal layer and enchondral ossification [19].
- Audiological findings: severe SNHL in IP-I; progressive hearing loss in IP-II; mixed HL or profound SNHL in IP-III.
- Management: patients who present IP-I malformation are usually suitable candidates for CI, while patients with IP-II may necessitate CI. Patients with IP-III presenting with moderate to severe mixed or SNHL can be managed with hearing aids. IP-III patients with severe HL are candidates for CI.

- Possible complications: Spontaneous cerebrospinal fluid fistula and recurrent meningitis can be seen in both IP-I (characteristic) and IP-II (less frequently) due, respectively, to a defect in the cribriform area between the cochlea and IAC and to a defective stapes footplate and modiolar base abnormalities [21, 29]. On the other hand, IP-III cases always have a high-volume CSF gusher during CI surgery but meningitis is very rare since the stapes footplate is normal.

### Enlarged vestibular aqueduct

The vestibular aqueduct is a structure of the IE being part of the osseous labyrinth. It contains the endolymphatic sac and duct and runs from the vestibule to the posterior cranial fossa.

EVA syndrome is characterised by enlarged vestibular aqueduct with SNHL. Pathological mechanism underling EVA syndrome is related to mutation in the SLC26A4 gene, which encodes for the pendrin protein. However, many other hypotheses have been proposed, including events that cause the variation of cerebrospinal fluid pressure [30]. HRCT is the choice of imaging modality to diagnose EVA [31]. However, MR imaging can give additional information about inner ear fluid status, which CT cannot provide.

**Fig. 11:**
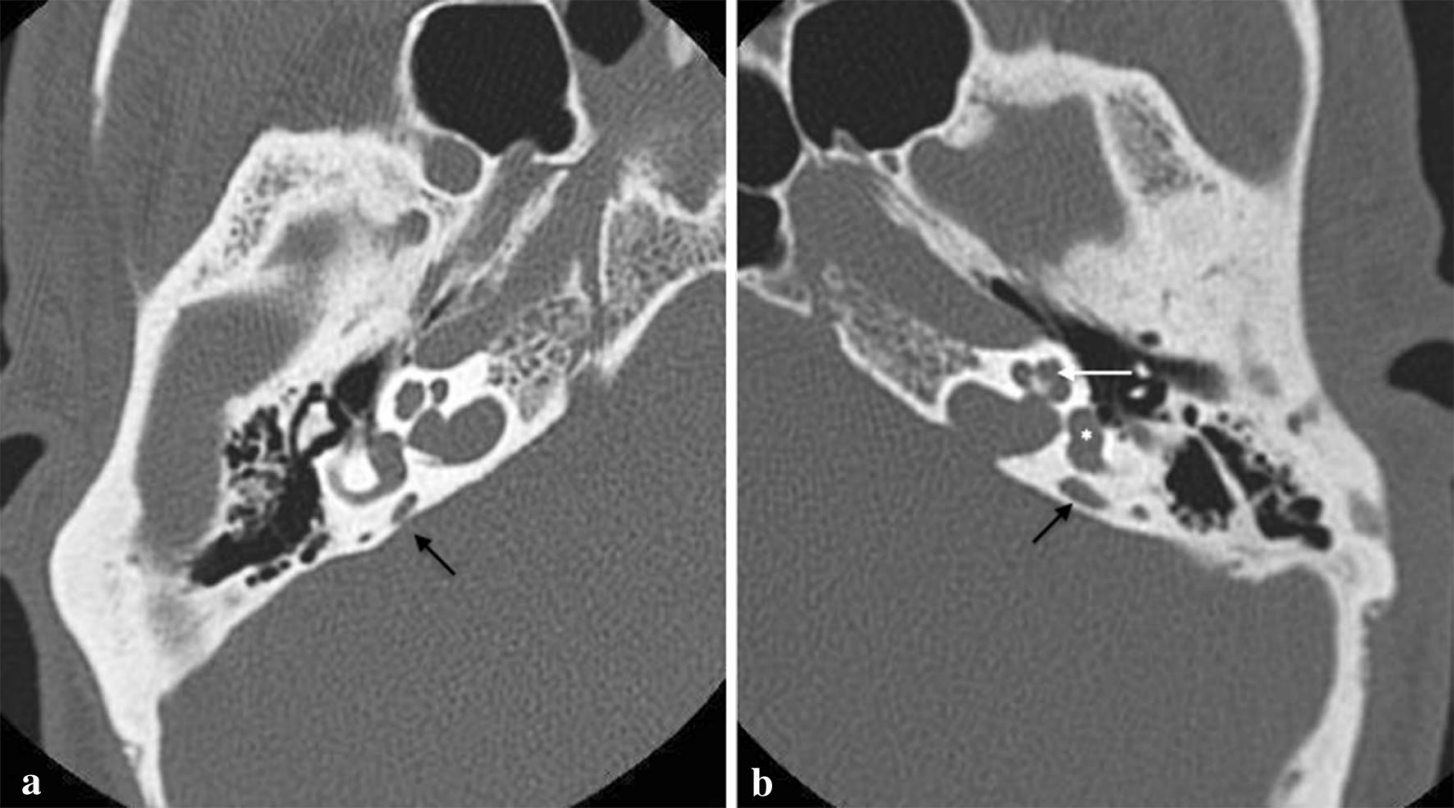
Enlarged vestibular aqueduct (EVA). A 7-year-old female with progressive bilateral SNHL. Axial CT obtained at IAC level show bilateral EVA syndrome. The cochlea (white arrow in **b**), the vestibule (asterisk in **b**) and SCCs have a normal appearance but there is a large vestibular aqueduct (black arrows in **a** and **b**)

**Fig. 12:**
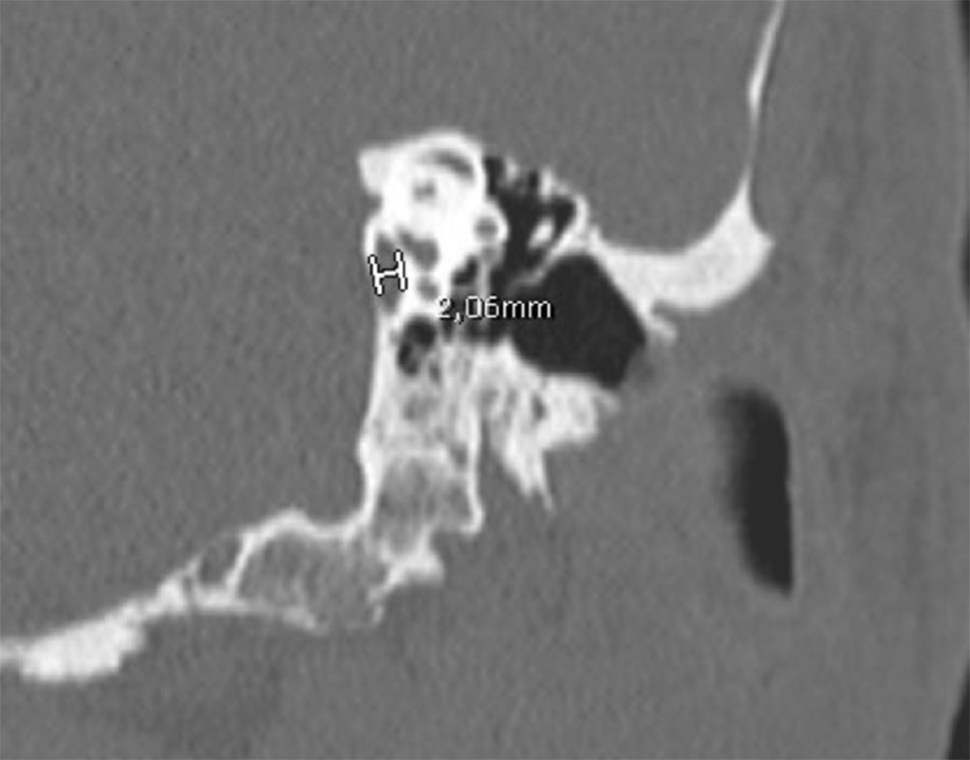
Same patient shown in Fig. 10. Multiplanar reconstruction on the coronal plane allows the vestibular aqueduct dilatation to be easily measured (> 1.5 mm is considered pathological)

According the Valvassori criteria, vestibular aqueduct is considered enlarged if: “vertical and axial width larger than 1.5 mm on the midpoint between labyrinth and operculum” (Figs. 11, 12) [32]. However, Cincinnati criteria (VA larger than 0.9 mm at the midpoint or larger than 1.9 mm at the operculum), proposed by Boston et al. in 2007, have been showed to be more sensitive to diagnose EVA [31–33].

When observed by CT and MRI, EVA is accompanied by a normal cochlea, vestibule, and SCCs [23]. This helps to differentiate EVA from IP-II [23].

#### Key points

- Audiological findings and surgical management are similar to IP-II.

### Malformations of the semicircular canals and vestibule

There is a wide variety of malformations of the SCCs and vestibule.

**Fig. 13:**
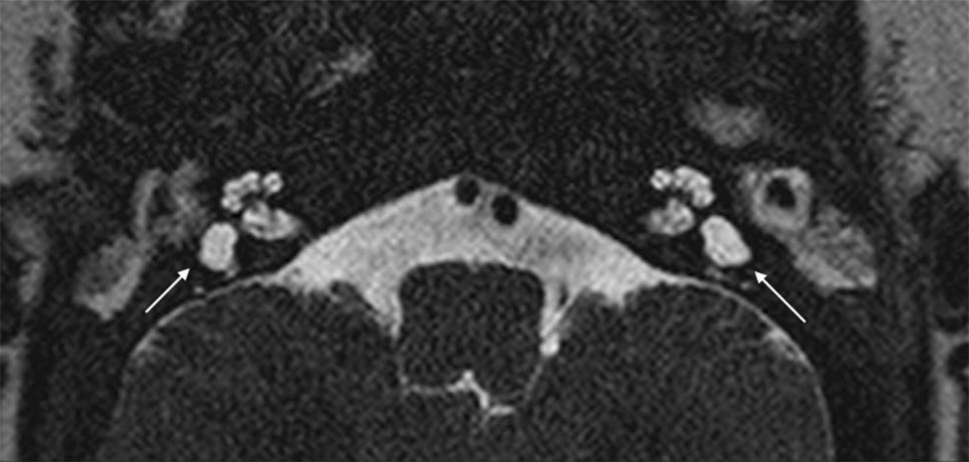
A 2-year-old male with CHARGE syndrome and bilateral SNHL. Axial 3D FIESTA sequence obtained at IAC level shows lateral SCCs-vestibule dysplasia (arrows) on both sides

The malformed canals may be short, wide, or narrow (Figs. 5, 13). Dysplasia of the SSCs is more frequent than aplasia. The absence of all semicircular ducts with normal cochlea occurs commonly in CHARGE syndrome [33–36], while patients with Waardenburg syndrome and Alagille syndrome show isolated aplasia of the posterior semicircular duct [33]. CT scans are important for corroborating the diagnosis of SCC aplasia and differentiating it from fibrous or calcified obliteration of the canals.

**Fig. 14:**
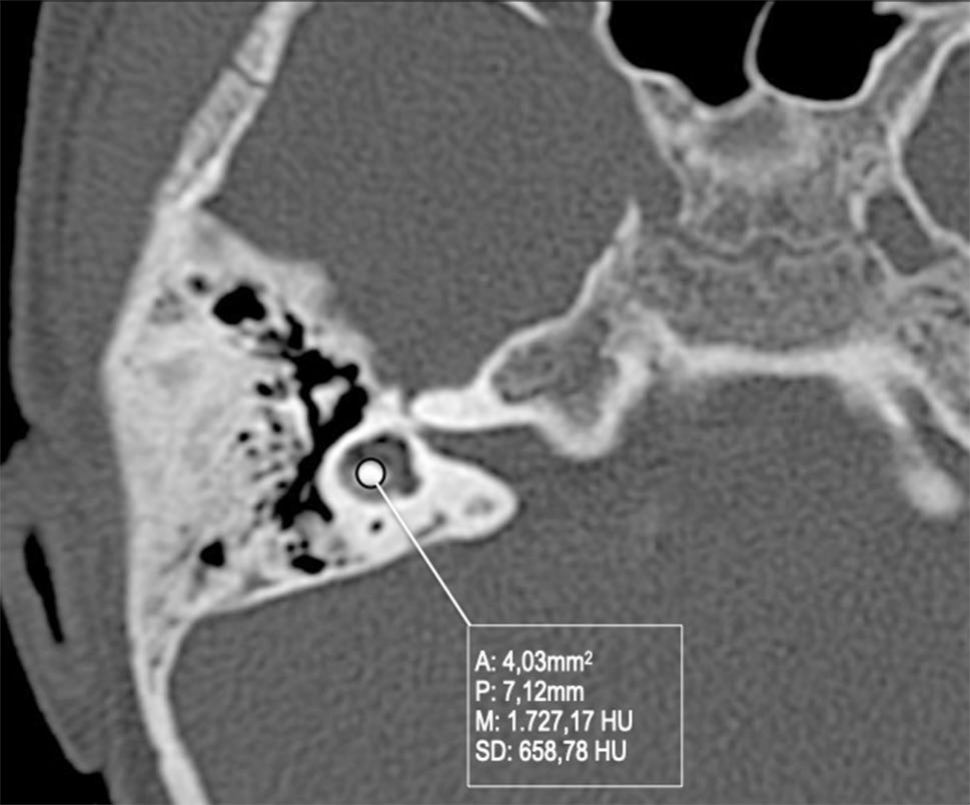
A 3-year-old female with right progressive SNHL. Axial CT obtained at IAC level shows an enlarged vestibule. Note that the bone island of the lateral SCC is below 6mm^2^ (measured 4.03 mm^2^)

Vestibule and utriculo-saccular anomalies are commonly found in relationship with other IEMs; isolated enlargement of the vestibule is more unusual (Fig. 14). Moreover, in the more complex malformations, the vestibule could form a CC with the lateral canal, accompanied by variable degrees of cochlear abnormalities.

#### Key points

- FN: a hypoplastic or aplastic lateral SCC leads to superior displacement of the tympanic segment of the FN;
- The vestibule can be considered enlarged if the CT shows that the area of the bone island surrounded by the SCC is less than 6 mm^2^ [26].

### Abnormalities of the vestibulocochlear nerve

**Fig. 15:**
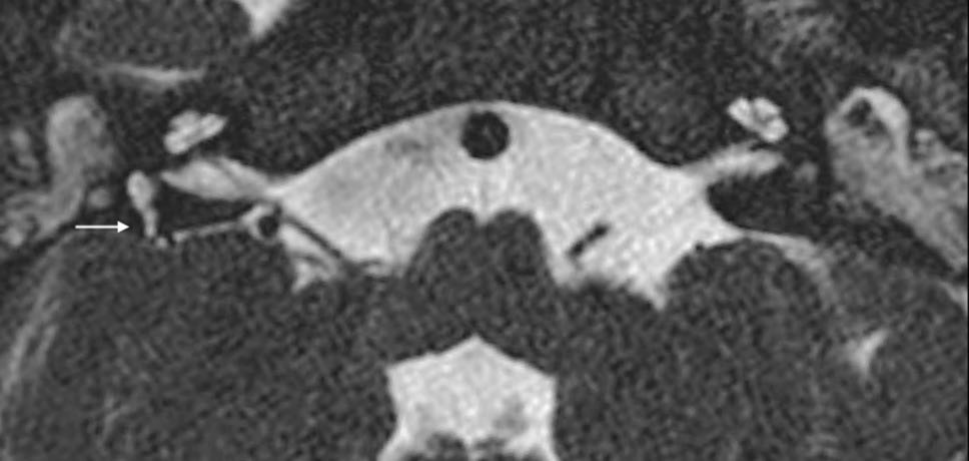
A 3-year-old female with CHARGE syndrome and bilateral SNHL from birth. Axial 3D FIESTA sequence obtained at IAC level shows right facial nerve hypoplasia and left cochlear nerve aplasia. Lateral SCCs dysplasia (arrow) and aplasia (left side) are also detected

**Fig. 16:**
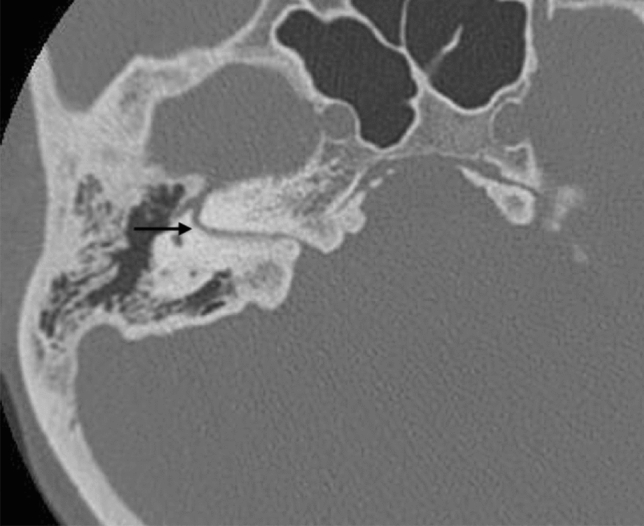
A 34-year-old male with IAC partition occasionally found in trauma screening. Isolated facial canal is demonstrated (arrow)

**Fig. 17:**
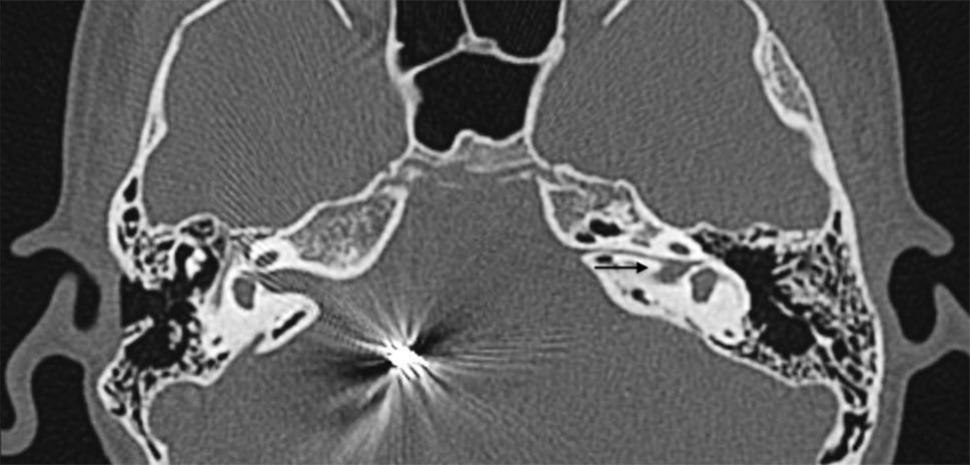
A 8-year-old female with bilateral complete HL since birth. Axial CT shows IAC partition on the left side characterised by an independent bony canal for the facial nerve that runs anteriorly (arrow) and an IAC that runs posteriorly (asterisk) populated by a hypoplastic cochlear nerve (not appreciable in CT imaging). Artefacts due to ABI on the right emi-pons in IP-II malformation of the same side are also appreciable

Current MRI techniques make it easy and simple to visualise and study the vestibulocochlear nerve [14]. The nerve may be normal to hypoplastic or absent (Fig. 15). The cochlear nerve is typically absent in cochlear aplasia cases (Fig. 4), while in the case of Michel deformity with absent IAC, the complete vestibulocochlear nerve is also absent and only the FN can be identified. IAC atresia or partition with hypoplasia to aplasia of the VIII cranial nerve can also be present without inner anomalies (Figs. 16, 17). A hypoplastic vestibulocochlear nerve is particularly important in CC. Indeed, the amount of fibres establishes the hearing level and management approach.

#### Key points

- Audiological findings: Severe to profound SNHL is usually present.
- The amount of cochlear nerve fibres determines the hearing level and management strategy (hearing aids, CI or ABI).

## Conclusion

HRCT and MR imaging are essential techniques for the assessment of IEMs. Since prompt intervention has a positive impact on the treatment outcomes, early diagnosis of IEMs can have a significant impact on the management and prognosis of these patients. A schematic approach to diagnosis of IEMs is presented in.

## Abbreviations

IE: Inner ear
SCCs: Semicircular canals
IAC: Internal acoustic canal
IEMs: Inner ear malformations
SNHL: Sensorineural hearing loss
CLA: Complete labyrinthine aplasia
CA: Cochlear aplasia
CADV: CA with a dilated vestibule
CC: Common cavity
CI: Cochlear implant
CH: Cochlear hypoplasia
IP: Incomplete partitions
EVA: Enlarged vestibular aqueduct
